# Addenda to the Report of a Case of Scurvy*See Lancet and Clinic, October 20, 1883

**Published:** 1883-12

**Authors:** Joseph Eichberg

**Affiliations:** Professor of Physiology, Miami Medical College, and Pathologist to Cincinnati Hospital.


### Addenda to the Report of a Case of Scurvy.*

BY JOSEPH EICHBERG, M. D.

Professor of Physiology, Miami Medical College, and Pathologist to Cincinnati Hospital.

Through the kindness of Dr. Philip S. Wales, Surgeon-General, U. S. N., I have come into possession of the annual reports issued by him during the last four years, and find therein some very valuable information with reference to the subject of the essay in the last number of the *Lancet and Clinic*. In commenting upon the question of rations for the navy, the Surgeon-General quotes the report of Surgeon Edward Kershner, for many years attached to the training-ship Minnesota, who says, after referring to the diet as established by acts of Congress: "It appears to have been decided upon after mature deliberation, and with due regard to the requirement of the naval service, but without, of course, taking into consideration the present youthful element,

* See *Lancet and Clinic*, October 20, 1883.

which composes the crews of the training-ships. This element requires a modification of old forms in various ways, and, probably, in none more than in the matter of diet.

On the training-ships we receive boys at the age of fifteen years; we have, therefore, to deal, not with the matured man, but with youth, and the conditions are very different, for here we have to supply material not only to keep the frame up to a normal standard, but to build up the boy into the able-bodied man.

The navy ration gives $6\frac{1}{2}$ pounds of bread and $6\frac{1}{2}$ pounds of meat a week, with a pound of flour. The British soldier, on home service, receives $5\frac{1}{4}$ pounds of meat and 7 pounds of bread, and buys additional bread, vegetables, and groceries, out of his pay. The Prussian soldier gets weekly 11 pounds 1 ounce of rye bread, and nearly $2\frac{1}{2}$ pounds of meat; and in the French infantry each man during peace gets weekly 15 pounds of bread, $3\frac{3}{10}$ pounds of meats, with some vegetables and a little brandy. They are expected to make up deficiencies from private means.

It will be noticed that the bread, in all these rations, exceeds that given in the United States Navy; and in that of France it is more than twice our allowance. It is permitted that the United States Navy ration may be changed so that fresh meat or fresh bread may be substituted for salt meat or biscuit; and the dried vegetables might be set aside and fresh substituted in their place.

A comparison of the schedules of rations of the European governments and my own experience warrant me to recommend a small increase in the ration of bread. I would further suggest the addition of hominy, oat meal, and wheat grits, to be used alternately at breakfast, which now consists mainly of coffee and bread. This would, probably, require a small additional increase of sugar or molasses to make these articles palatable.

Salted fish and cheese are articles of a wholesome character, and quite nutritious.

The dried potatoes are very rarely used, as the men have a dislike for them. It would be an improvement to issue in their place, whenever it is possible, green potatoes. Other vegetables issued in season would also promote the health of the boys—such as turnips, onions, carrots, beets, tomatoes, and sweet potatoes. Apples, cresses, etc., would be useful.

The neglect of vegetables in the navy is, in a physiological point of view, imprudent, and may be a cause of many of the digestive derangements from which sailors suffer, and, possibly, a contributing cause of an inordinate thirst for alcohol, which characterizes and impoverishes so many sailors. To satisfy their instinctive craving for a hydro-carbon, they take one convenient indeed, in some respects, but of which an excess is very unwholesome.

Experience, too, tends to show that the raising of the energies to their full height of usefulness may be effected by vegetable food quite as well as by the more stimulating and expensive animal nutriment.

A varied, plentiful, and healthy diet for its servants, will always be a good and economical investment for any government, as it promotes effective, cheerful, and efficient discharge of duty, which is always remunerative."

The report of Surgeon J. H. Kidder, states:

"According to an exhibit published by the Paymaster-General of the Navy, June 1, 1872, and now followed in vessels of war at sea, the daily distribution of the ration is as follows:

*Sunday*—14 oz. biscuit; $\frac{3}{4}$ lb. preserved meat; 8 oz. rice; 4 oz. sugar; 0.57 oz. butter; 2 oz. dried potatoes; 1.14 oz. molasses.

*Monday*—14 oz. of biscuit; 1 lb. salt pork; 8 oz. beans; 4 oz. sugar; 0.57 oz. butter; 1.14 oz. molasses.

*Tuesday*—14 oz. biscuit; 1 lb. salt beef; 8 oz. flour; 2 oz. dried fruit; 4 oz. sugar; 0.57 oz. butter; 1.14 oz. molasses.

*Wednesday*—14 oz. biscuit; 1 lb. salt pork; 4 oz. sugar; 8 oz. beans or peas; 0.57 oz. butter; 1.14 oz. molasses.

*Thursday*—14 oz. biscuit; $\frac{3}{4}$ lb. preserved meat; 4 oz. sugar; 2 oz. dried potatoes; 0.57 oz. butter; 1.14 oz. molasses.

*Friday*—14 oz. biscuit; 1 lb. salt beef; 8 oz. flour; 4 oz. sugar; 8 oz. dried fruits; 0.57 oz. butter; 1.14 oz. molasses.

*Saturday*—14 oz. biscuit; 1 lb. salt pork; 4 oz. sugar; 8 oz. beans; 0.57 oz. butter; 1.14 oz. molasses.

One-half oz. of tea, or 2 oz. coffee, issued daily; $1\frac{1}{2}$ pints vinegar and 2 oz. pickles, issued weekly; 1 lb. fresh bread, or 1 lb. flour, or $\frac{1}{2}$ lb. rice, may be substituted for 14 oz. biscuit; $\frac{1}{2}$ lb. rice for $\frac{1}{2}$ lb. beans or peas, or vice versa. Fresh or preserved meat may be substituted for salt in the proportion of $1\frac{1}{4}$ lbs. fresh, or $\frac{3}{4}$ lb. preserved meat, to 1 lb. of salt meat; and vegetables for the other articles in the list (besides meat) to the extent of their money value. Two rations in ten may be commuted."

At the close of his very exhaustive article, in which he proves that the albuminous and fatty principles are chiefly deficient in this ration for growing boys, Dr. Kidder calls attention to the following points in connection with them:

1. They get but one hot and sufficiently varied meal a day (dinner).

2. They get a cold breakfast, consisting of a single food element—dry bread. Fats are naturally called for at breakfast to start the machinery of digestion.

3. All three of their meals are crowded into about nine hours, leaving fifteen hours of fasting. Growing boys should be fed often, and the younger they are the oftener they should be fed.

4. The large quantity of coffee consumed daily, without a corresponding supply of solid food to accompany it, is likely to give rise to atonic dyspepsia.

5. Is it not possible to improve the present mode of cooking the ration, so that more of the fatty parts, now wasted as "slush," may be saved as food and greater variety in form obtained? The French navy appears to have met with fair success in this direction."

In the report for 1880 Dr. Kershner says:

"Considerable complaint has been made by the boys at various times about the food. Five rations in every mess of 20 boys have been commuted, and the money spent in buying articles not on the supply table. A greater variety of articles were thus obtained, and messes were supplied with oat-meal, milk, and various vegetables, as well as poultry and meats. In a letter to the department in 1879, I stated that the ration was insufficient in some respects. I do not now think the remedy has been reached by the commutation of rations and purchase of provisions with the money, or that the nutritive equivalent of the navy ration can be purchasrd by money obtained by its commutation. I therefore think it necessary that the commutation price of the ration be increased, or that articles not now in the ration be allowed. Especially should potatoes, tomatoes, onions, corn (green or canned), oat-meal, corn-meal, hominy, cheese, and a larger quantity of bread, fresh meat, and sugar, in some form be added. If such changes could be made it would be of great value to the boys, promoting their physical development, rendering them more contented, enabling them to endure the hardships of a sailor's life."

A very able and interesting circular was issued by the Bureau of Medicine and Surgery in 1880 to the Rodgers, which left San Francisco in June of that year to search for the unfortunate Jeannette, about which considerable anxiety was felt at the time. This circular bears the title, "Sanitary Instructions for the Information and Guidance of the Jeannette Search Expedition," and embraces as complete a list of prophylactic measures against scurvy as it is possible to find. The regulations embodied in the report embrace all of the more modern advances in hygiene, and give very instructive information as to the preparation and value of certain articles of diet. In the course of the circular we find the following: "*One of the most valuable anti-scorbutic substitutes for fresh succulent vegetables is lime juice*, which should be supplied the men daily in the quantity of one ounce mixed with an equal weight of sugar. It is recommended that the issue of lime juice begin after leaving port, when the supplies of fresh vegetables have been exhausted, and that an officer should see that each man has taken the prescribed quantity. This last suggestion is important, since, if it is left to the individual caprice or fancy, it may be neglected." And, again, "The *indispensable necessity of lime juice* in the sledging parties, and the difficulties of carrying and preparing it for use, induced me to suggest the propriety of combining the juice and pemmican in the proportion of one ounce to the pound of the latter. A large quantity of this preparation has been furnished for use in the sledging parties, and it is believed that great advantage will accrue from it. The pemmican is greatly improved in taste and flavor, and will, I believe, be more assimilable. This is an important modification, as there are persons who cannot eat the ordinary article."

The want of space, unfortunately, prevents our reproducing here the whole of this most valuable circular, containing, as it does, instructions for ventilation, clothing, the preparation of and kind of diet, the amount of labor to be done, and the precautions necessary to prevent the more common accidents likely to occur in a polar climate. The Rodgers, as is well known, set sail in June, 1880, remaining in service 123 days, and was destroyed by fire at St. Lawrence Bay, November 3rd, of the same year. In the Surgeon-General's report for 1881, we find it stated that "she was remarkably exempt from disease during this service, only four cases having been reported, and these of a trivial character." The assistant surgeon of the vessel, J. D. Castillo, appends his report, from which the following selections bear directly upon our subject:

"No cases of scurvy occurred before the 25th of January, 1882, that is, 56 days after the ship was burnt, during which time, no doubt, the peculiar agencies which are supposed to favor the appearance of scurvy were steadily operating on our men. Those attacked were by no means the weakest among our crew, nor were they intemperate men, with the exception, may be, of two, who, however, did not suffer severely. Whatever the causes of scurvy may be, in this case the poor hygienic surroundings played a most important part in the causation of the disease. If we take into consideration that a body of men, already more or less fatigued by a long cruise, were thrown entirely out of their usual mode of living, not only deprived of their accustomed articles of diet, but also compelled to live in filthy houses, without ventilation, and only artificially lighted, we cannot wonder at the outbreak of scurvy, and must only feel surprised that the disease presented so mild a character.

The first case that came under my notice was that of Mr. De T., ship's carpenter, who reported to me, January 22nd, that his gums were sore, and that he had been spitting mouthfuls of blood during the day and night. The blood was oozing principally from around the second molar tooth on the left side of the upper jaw. All attempts to stop the bleeding proved futile, snow at several degrees below zero being used to no purpose. Next morning, the hemorrhage still continuing unabated, and the patient becoming considerably debilitated, I found him looking very pale, his complexion sallow, the sallowness being most marked over the forehead and cheeks; his gums were spongy, presenting a granular appearance, and were already becoming detached from the neck of the teeth; their color was also altered; they had become purplish, and in some places almost black. The only styptic at my command being the actual cautery, I resolved to cauterize the bleeding surface with a button-hook heated to a red heat over a seal-oil flame. While heating the instrument, and when this was almost ready, De T. suddenly announced that the bleeding had ceased, probably from nervous shock. Some powdered alum was afterwards used, and the gums were painted with tincture of iodine twice a day for the first two or three days, and there was no recurrence of the hemorrhage; the gums, however, continued sore for some time, and bled slightly whenever the patient chewed food. He was put upon one ounce of lime-juice three times a day, and was also to take three doses of citric acid, five grains to a dose. On the first of February his condition was found as follows: Gums sore and spongy; no recurrence of the hemorrhage; pain in the long bones; disinclination to exercise; great despondency and emaciation; slight diarrhœa; shortness of breath; but no scurvy spots to be seen in any part of the body.

Shortly afterwards some three other men complained of sore and bleeding gums, and they were put upon the same treatment. Their improvement was slow, probably from unfavorable surroundings, and especially mental influences, but it was more rapid towards spring, when the less severe weather enabled the patients to exercise more in the open air and enjoy the light of the sun.

On the 20th of March I was relieved by Dr. Jones, and went up to the post, established by Captain Berry on the Northern coast, so that I lost sight of Mr. De T. until I saw him again on the 10th of May, when I found him almost well. The gums, however, remained slightly spongy, and the body was somewhat wasted; he had, however, regained his good spirits, as he was already on board a whaler, with civilized clothes on, and eating hard bread and molasses.

The next case was that of H. S. W., master; this patient presented some acute symptoms, and the disease was directly traceable to *overwork, exposure, and fatigue, as the exciting cause.* After the disappearance of Master C. F. Putnam, who was blown off shore on an ice floe on the 11th of January, Mr. W. undertook a series of sledge-journeys in search of him, thoroughly examining the coast for a distance of 200 miles. He was away upwards of a month, and at his return looked completely broken down, having been travelling most of the time since he left us, and obliged to sleep out on several occasions on account of gales of wind and snow-drifts. These hardships, operating on a constitution already weakened by confinement and privation, contributed to the appearance of the disease. On the 24th of February four or five days after his return, Mr. W. first complained of spitting blood, and thought he was going to have an abscess around one of the upper molar teeth. Upon examination his gums were found very spongy; they bled when but slightly touched by the finger, and were becoming detached from around the neck of the teeth; a brownish line was also to be seen at the edge of the gums, which was, no doubt, formed by necrotic tissue, imparting great fetor to the breath. The patient also complained of lassitude; had lost a great deal of flesh, and become very sallow. He was immediately put upon 3 ounces of lime-juice, and 15 grains of citric acid a day.

Feb. 28—Mr. W. not improving, the gums continue very sore and bleeding; feels weaker, and has great aversion to exercise.

March 2—Patient complains of sore throat; on examination tonsils were found perfectly normal, but on the left anterior column of the fauces is to be seen a red patch, ecchymotic in nature; the tissues around are tumefied, causing painful deglutition. The patient feels very despondent; he is very peevish and will not leave his hut. All attempts to cheer him up are vain, and thinks he is going to die. Over his legs, around the ankle and calf especially, are to be seen purple, ulcerated spots.

March 6--The tumefaction of the tissues of the fauces had diminished; deglutition easier; no change in the condition of the gums; losing flesh rapidly; spirits very low.

March 8--Worse. The right anterior column of the fauces presents an ecchymotic patch; skin of face and popliteal spaces very sallow; breath very fetid; marked dyspnœa on exercising; no congestion of the lungs noticeable.

March 9--Throat less sore; other symptoms about the same.

March 10--Throat much better; sense of lassitude diminishing; patient more cheerful.

March 11—Throat almost well; general improvements. From this date Mr. W. steadily improved; the only symptom which remained unabated was the sponginess of the gums, which were still in a pretty bad condition when the patient reached San Francisco, after enjoying a month of good, varied, fresh diet.

March 16--Throat well; no soreness left, but ecchymotic patches still present; patient feels better in every respect; takes pleasure in exercising, and is able to keep longer out of doors, as the weather is already quite mild.

On the 20th of March I lost sight of the patient, and on the 10th of May, when I saw him again, I found him greatly changed for the better; he was then very cheerful and felt strong enough to make a sledge journey of 30 miles three days in succession.

Here we have two cases of scurvy, both presenting about the same symptoms, both treated with lime-juice; in one we witness a speedy recovery, or at least amelioration; the other remains stationary, and seems only to be benefitted by the influence of spring and the return of the sun. Perhaps an explanation of the apparent failure of lime-juice in Mr. DeT.'s case may be found in the fact already mentioned, that his surroundings were not calculated to keep him in a cheerful mind, being always in a state of mental anxiety, fearing violence at the hands of the natives, and always thinking of his wife and children, who, should he die, as he often said, would be left without a support in this world.

The other men who suffered with scurvy presented such mild symptoms, that no separate record was kept of them; the principal symptoms were bleeding gums, sallowness of the complexion, and a general sense of lassitude. No lime-juice was given them, as it could not be spared, and was being used for the two severer cases already given.

Our crew consisted of 28 persons, 6 of whom, that is, over 20 per cent. suffered with scurvy in a lesser or greater degree."

This is, probably, the last epidemic of scurvy in the history of polar navigation. The history of the cases is of great interest when compared with our own, and the predisposing causes are clearly set forth. It is not necessary to comment upon what has already been discussed at considerable length in the previous article, and I can only regret that want of space should prevent my reproducing the circular letter already referred to. In a polar expedition the possibility of an outbreak of scurvy is constantly present to the mind, and prophylactic measures are resorted to with a view to its prevention. In private practice we are, however, not accustomed to look for the disease, and must, therefore, not lose sight of it in arriving at the diagnosis.—*Lancet and Clinic.*

